# Impaired cellular bioenergetics caused by GBA1 depletion sensitizes neurons to calcium overload

**DOI:** 10.1038/s41418-019-0442-2

**Published:** 2019-11-04

**Authors:** Nicoletta Plotegher, Dany Perocheau, Ruggero Ferrazza, Giulia Massaro, Gauri Bhosale, Federico Zambon, Ahad A. Rahim, Graziano Guella, Simon N. Waddington, Gyorgy Szabadkai, Michael R. Duchen

**Affiliations:** 1 Cell and Developmental Biology Department, University College London, London, WC1E6XA UK; 2 Institute for Women’s Health, University College London, London, WC1E6HU UK; 3 Department of Physics, University of Trento, 38123 Povo (TN), Italy; 4 School of Pharmacy, University College London, London, WC1N1AX UK; 5 MRC Antiviral Gene Therapy Research Unit, Faculty of Health Sciences, University of the Witwatersrand, Johannesburg, South Africa; 6 Department of Biomedical Sciences, University of Padua, 35131 Padua, Italy; 7 The Francis Crick Institute, London, NW1 1AT UK; 8 Present Address: Department of Biology, University of Padua, 35131 Padua, Italy

**Keywords:** Neuroscience, Physiology, Neurological disorders

## Abstract

Heterozygous mutations of the lysosomal enzyme glucocerebrosidase (*GBA1*) represent the major genetic risk for Parkinson’s disease (PD), while homozygous *GBA1* mutations cause Gaucher disease, a lysosomal storage disorder, which may involve severe neurodegeneration. We have previously demonstrated impaired autophagy and proteasomal degradation pathways and mitochondrial dysfunction in neurons from *GBA1* knockout (*gba1*^*−/−*^) mice. We now show that stimulation with physiological glutamate concentrations causes pathological [Ca^2+^]_c_ responses and delayed calcium deregulation, collapse of mitochondrial membrane potential and an irreversible fall in the ATP/ADP ratio. Mitochondrial Ca^2+^ uptake was reduced in *gba1*^−/−^ cells as was expression of the mitochondrial calcium uniporter. The rate of free radical generation was increased in *gba1*^−/−^ neurons. Behavior of *gba1*^*+/−*^ neurons was similar to *gba1*^*−/−*^ in terms of all variables, consistent with a contribution of these mechanisms to the pathogenesis of PD. These data signpost reduced bioenergetic capacity and [Ca^2+^]_c_ dysregulation as mechanisms driving neurodegeneration.

## Introduction

The *GBA1* gene encodes for the lysosomal enzyme glucocerebrosidase (GBA1), which hydrolyzes the lipid glucosylceramide [1]. Homozygous mutations in *GBA1* cause an inherited lysosomal storage disease known as Gaucher disease (GD) [2], while heterozygous mutations in *GBA1* are the major known genetic risk factor for Parkinson’s disease (PD) [3–5]. GD presents with a spectrum of symptoms and is classified into three ‘types’: [6] while type I shows limited or late CNS involvement, type II presents with a severe and progressive neurodegeneration, and type III with less severe, chronic neurological symptoms. PD signs and symptoms include resting tremors, bradykinesia, and rigidity; anosmia, depression, and anxiety, and at later stages, dementia. The links between specific GBA1 mutations and phenotype remain unclear [6, 7].

Both PD and lysosomal storage diseases such as GD are characterized by dysfunction of the autophagy/lysosomal pathway and impaired mitochondrial function [8, 9], suggesting that deficiencies in each of these pathways can affect the other [10]. We have previously shown that, in neurons from *gba1*^*−/−*^ mice, autophagy is impaired upstream of the lysosome, associated with profoundly impaired mitochondrial function, decreased mitochondrial membrane potential (Δψ_m_), reduced mitochondrial respiration, and especially a dramatic decrease in uncoupled maximal respiratory capacity [11]. Interestingly, *gba1*^*+/−*^ neurons showed a modest reduction in Δψ_m_, reflecting the absence of symptoms in the *gba1*^*+/−*^ mice compared with *gba1*^*−/−*^ [12].

The mechanism linking mitochondrial dysfunction to the underlying primary defect in lysosomal biology remains unresolved but may be attributable to the accumulation of dysfunctional mitochondria due to impaired mitochondrial quality control pathways. This has also been described in other lysosomal storage diseases (reviewed in [8]).

Mitochondria show an intimate and complex relationship with calcium signaling, representing a major intersection between cellular bioenergetics and cell signaling pathways [13–15]. Mitochondria take up Ca^2+^ from the cytosol, in a process mediated by the mitochondrial Ca^2+^ uniporter (MCU) complex [16], its regulatory proteins [17–19], and balanced by the mitochondrial Ca^2+^ efflux pathway [20].

Dysregulation of [Ca^2+^]_c_ signaling has been implicated widely in neurodegeneration and has been identified as a key pathway in the selective degeneration of dopaminergic neurons in PD [21, 22]. We therefore explored the interplay between [Ca^2+^]_c_ homeostasis and mitochondrial bioenergetic capacity as potential contributors the neurodegeneration associated with *GBA1* depletion, studying [Ca^2+^]_c_ signaling in primary neuronal cultures from *gba1*^*−/−*^, *gba1*^*+/−*^, and *gba1*^*+/+*^ mice, stimulated with glutamate at physiological concentrations that are innocuous to control neurons (10 μM or below). As Ca^2+^-dependent mitochondrial dysfunction is exacerbated by oxidative stress [15, 23], we also explored changes in free radical production associated with *GBA1* depletion.

We found that in both *gba1*^*−/−*^ and *gba1*^*+/−*^ neurons, exposure to physiological glutamate concentrations caused delayed calcium deregulation (DCD), loss of Δψ_m_ and bioenergetic failure. Intriguingly, MCU protein expression was downregulated in *gba1*^*−/−*^, associated with a reduced mitochondrial Ca^2+^ buffering capacity.

Very importantly, *gba1*^*+/−*^ neurons, which model to a certain extent the heterozygous *GBA1* mutations found in PD patients, behaved similarly to *gba1*^*−/−*^.

These findings emphasize the fundamental importance of mitochondrial bioenergetic capacity in maintaining neuronal energy homeostasis in the face of increased energetic demand associated with activity. Our data demonstrate the vulnerability of neurons in which mitochondrial function is perturbed to cellular [Ca^2+^]_c_ overload, suggesting that increased sensitivity to physiological glutamate concentrations may play an important role in neurodegeneration in GD and possibly also in GBA1-related PD.

## Methods and materials

### Mouse model and animal welfare

Mice used for this work were described in Enquist et al. [12]. As K14-wt, K14-lnl/wt (lox/neomycin/lox), and K14-lnl/lnl and are herein referred as *gba1*^*+/+*^, *gba1*^*+/−*^, and *gba1*^*−/−*^.

Mouse welfare was approved by the University College London Animal Welfare and Ethical Review Board (AWERB) and in accordance with personal licenses granted by the UK Home Office and the Animal (Scientific Procedures) Act of 1986. The colony was maintained using breeding pairs heterozygous for GBA1 and pups were euthanized at P0 using cervical dislocation followed by decapitation for primary neuronal cultures preparation and liver extraction for genotyping.

### Mouse genotyping

Genomic DNA was extracted from liver for each pup (P0) using DNeasy Blood & Tissue Kit (Qiagen), genotyped by PCR using Q5 High Fidelity DNA Polymerase (NEB) and the following primers:

**Table:** 

GCex8-2 (Sigma, USA)	GTACGTTCATGGCATTGCTGTTCACT
METex8-2 (Sigma, USA)	ATTCCAGCTGTCCCTCGTCTCC
NEO-AO2 (Sigma, USA)	AAGACAGAATAAAACGCACGGGTGTTGG

PCR was performed on T100 Thermal cycler (Biorad) using the following PCR cycling conditions. Bands were visualized by gel electrophoresis.

**Table:** 

98 °C	30 s	×15
98 °C	10 s	
63 °C (0.5 °C touchdown)	30 s	
72 °C	1.30 min	
98 °C	10 s	×25
61 °C	30 s	
72 °C	1.30 min	
72 °C	5 mins	
10 °C	Hold	

### Neuronal and astrocyte primary culture

Mixed cultures of cortical neurons and astrocytes were obtained by dissecting brains from P0-P1 mice. Cortices from each brain were isolated, kept in HBSS (H6648, Sigma) on ice separated from each other and genotyping was performed on liver extracted from each pup.

Brain tissue was incubated in EBSS (E2888, Sigma) and papain (LK003178, Worthington Biochemical Corp.) for 40 min at 37 °C and it was then dissociated by trituration in EBSS supplemented with DNAse (LK003172, Worthington Biochemical Corp.) and papain inhibitor (LK003182, Worthington Biochemical Corp.). After spinning, the cell pellet was resuspended in Neurobasal (21103-049, Life Technologies), supplemented with B27 (17504-044, Life Technologies), Glutamax (35050-038, Life Technologies), and 100 U/ml Penicillin–Streptomycin (1514-122, Life Technologies) counted and plated to appropriate densities on coverslips (0.5·10^6^ cells), 6-well plates (10^6^ cells) or 96-well plates (30000 cells), coated with polylysine (P4707, Sigma). Half media changes were done every 4 or 7 days. Cultures were maintained at 37 °C and 5% CO_2_ in humidified atmosphere and used between 10 and 15 days in vitro unless stated differently.

### Cytosolic calcium imaging

Primary neurons and astrocytes in mixed cultures were seeded onto 22-mm coverslips (or on 35mm FluoroDishes™ (Fisherscientific)) and stained with 5 μM FuraFF or Fura2 (F14181 and F1221, Thermo Fisher Scientific) in recording buffer (150 mM NaCl, 4.25 mM KCl, 4 mM NaHCO_3_, 1.25 mM NaH_2_PO_4_, 1.2 mM CaCl_2_, 10 mM D-glucose, and 10 mM HEPES at pH 7.4) with pluronic acid 0.02%, at 37 °C and 5% CO_2_ for 30 min.

After washing, cells were imaged in recording buffer using a custom-made imaging widefield system built on an IX71 Olympus microscope equipped with a 20× water objective. A Xenon arc lamp with a monochromator was used for excitation, exciting FuraFF or Fura2 fluorescence alternately at 340 nm ± 20 nm and 380 nm ± 20 nm and collecting emitted light through a dichroic T510lpxru or a 79003-ET Fura2/TRITC (Chroma), and a band-pass filter 535/30 nm. Images were acquired using a Zyla CMOS camera (Andor) every 2–4 s and neurons were stimulated using 10 μM glutamate (G1626, Sigma). A total of 2 μM ionomycin was added at the end of each time course as a positive control.

Electrical stimulation experiments were performed using a ‘myopacer’ (Ionoptix, Westwood USA) electrical stimulator and custom-made platinum electrodes (settings were: 40 V, 5 Hz 40 msec pulses and each stimulation period lasted 10 s).

Activation of metabotropic glutamate receptors was achieved by challenging neurons with 100 μM quisqualate (Q2128, Sigma), while inhibiting NMDA and AMPA/kainate receptors using 10 μM (2 R)-amino-5-phosphonopentanoate (D-AP5) (0106/100, Bio Techne Ltd) and 20 μM CNQX (HB0205, HelloBio), respectively.

Images were analyzed using ImageJ by selecting regions of interest (ROI) in each cell and measuring average fluorescence intensity in the ROIs for each channel. After background subtraction, ratios between the signal excited at 380 nm and at 340 nm were calculated at each time point and the resulting 340/380 ratioed traces representing cytosolic [Ca^2+^]_c_ levels upon stimulation were plotted. Peak amplitude values were calculated for each cell using Microsoft Excel and GraphPrism.

### Rhodamine123 imaging

Mixed cultures of primary neurons and astrocytes seeded on 22-mm coverslips were labeled with 10 μg/ml Rhodamine123 (R8004, Sigma) in recording buffer, at 37 °C and 5% CO_2_ for 20 min. After washing, cells were imaged in recording buffer using a Zeiss 880 confocal microscope equipped with a 40× oil objective, exciting neurons at 488 nm and collecting light longer than 505 nm. Images were acquired every 20 s with low laser power to avoid light-induced mitochondrial depolarization and photobleaching. Neurons were stimulated using 10 μM glutamate (G1626, Sigma) and 1 μM Carbonyl cyanide 4-(trifluoromethoxy)phenylhydrazone (FCCP) (C2920, Sigma) was added at the end of the time course to evaluate the Rhod123 fluorescence intensity corresponding to 100% depolarization. Images were analyzed using ImageJ by selecting ROI in each field and measuring average fluorescence intensity in the ROIs. For each trace, the signal was normalized between basal (the minimum value of intensity set to 0%) while the fully depolarized signal was set as the intensity of the maximal data point, representing complete depolarization, at 100%. Cumulative frequency distribution analysis was also performed as for the measurements of [Ca^2+^]_c_.

### Western blotting

Brains tissue dissection was performed at P0-P1 and samples were snap frozen in liquid nitrogen and stored at −80 °C. To extract soluble proteins, brain tissue was homogenized in RIPA buffer (150 mM NaCl, 0.5% Sodium deoxycholic acid, 0.1% SDS, 1% Triton X-100, 50 mM Tris pH 8.0, 1 mM PMSF (93482, Sigma)) and incubated 30 min on ice. After incubation, lysates were centrifuged and the soluble fraction was collected to quantify protein concentration by Pierce BCA Protein Assay Kit (23225, Thermo Scientific). Sample buffer 4× with 2% beta-meracapto ethanol was added to 30–50 ug of total proteins, samples were boiled and loaded onto a Nu-Page gel (4–12% or 12%) (NP0335 and NP0341, Invitrogen) using MOPS or MEF buffer (NP001 or NP002, Invitrogen). Gels were transferred to 0.45 μm PVDF membranes (IPVH00010, Millipore) in transfer buffer (NP0006, Invitrogen) with 20% methanol using a semi-dry system (Invitrogen).

After blocking in 5% milk T-TBS buffer (20 mM Tris, 150 mM NaCl pH 7.4, and 0.1% Tween 20), membranes were probed with primary antibodies (MCU, HPA016480 Sigma; EMRE, sc-86337 Santa Cruz; SOD1, sc-8637 Santa Cruz; SOD2, sc-137254 Santa Cruz; grp75, sc-1058 Santa Cruz; β-actin, A2228 Sigma; MICU2, ab101465 Abcam; MCURI, ab86335, Abcam; GRIN2b, AGC-003 Alomone labs; GRIK2, AGC-009 Alomone labs; Phospho-p40phox (Thr154), #4311 Cell Signaling Technology) overnight at 4 °C, washed and probed with an appropriate HRP-conjugated secondary antibody (anti-rabbit, 31463 Thermo Fisher Scientific; anti-mouse, 31457 Thermo Fisher Scientific; anti-goat, A5420 Sigma) for 1 h at room temperature. Visualization was performed using Luminata Forte Western HRP substrate (WBLUF0100, Millipore) and Chemidoc imaging system (Biorad).

### ATP:ADP measurements using PercevalHR and confocal imaging

A total of 30,000–50,000 neurons were plated in 96-well plates with clear bottom and transfected with the ATP:ADP sensor PercevalHR [24] at DIV8 using Lipofectamine LTX (15338-030, Life Technologies) in Optimem (31985-047, Life Technologies) for 1 h. Conditioned media were kept and used to replace Optimem after transfection. After 48–72 h, imaging of single neurons expressing PercevalHR was performed in recording buffer exciting PercevalHR at 405 nm and at 488 nm and collecting light at wavelengths longer than 510 nm, using a Zeiss 880 confocal microscope. Images were acquired every 10 s and neurons were stimulated using 10 μM glutamate (G1626, Sigma) at 200 s. ATP:ADP ratios over time were obtained drawing ROIs in each neuron, measuring average fluorescence intensity in the ROIs for each channel in ImageJ and calculating the 488 nm/405 nm ratio.

### Mitochondrial calcium measurement using mitochondrial-aequorin

A total of 30,000–50,000 mixed neurons and astrocytes were plated and grown onto white 96-well plates, transduced with mtAequorin adenovirus [25] at DIV7–8 and incubated for 48–72 h. Afterward, media was replaced with 5 µM coelenterazine in Krebs Ringer Buffer (125 mM NaCl, 5.5 mM D-Glucose, 5 mM KCl, 20 mM HEPES, 1 mM Na_3_PO_4_, 1 mM Glutamine, 100 mM Pyruvate, and 1.2 mM CaCl_2_). The plate was then incubated in the dark for 2 h at 37 °C. Luminescence measurements were obtained using a plate reader (FluoStar Optima, BMG Labtech) every 1 s after 10 µM glutamate stimulation at 5 s. A total of 100 µM Digitonin was added at 25 s. Mitochondrial Ca^2+^ concentrations were calculated as previously described [25].

### Measurement of rates of ROS generation

Mixed neurons and astrocytes plated on coverslips were imaged in recording buffer using a Zeiss 510 confocal microscope, equipped with a UV laser (Coherent). 5 μM dihydroethidium (DHE, D1168 Thermo Fisher Scientific) was added to recording buffer after starting the acquisition. Reduced DHE was excited at 351 nm and emitted light was recorded between 435 and 485 nm; oxidized DHE was excited at 543 nm and emitted light was collected using a 560-nm long-pass filter. Images were acquired over time every 8.93 s and 10 μM glutamate was added at 600 s to measure changes induced by glutamate stimulation in oxidation rates. Oxidation rate curves were obtained calculating the ratio between reduced and oxidized DHE. Basal oxidation levels (basal slopes) were calculated considering the difference between ratio at 500 s and the ratio at the beginning of the time course and dividing it by the time duration. Glutamate-induced oxidation rates were calculated as the difference between ratios at 1000 s and at 500 s and dividing it by the time duration (glutamate slopes).

### Measurement of glutathione levels using monochlorobimane (MBC)

Monocholorobimane (M1381MP, Invitrogen) imaging experiments were performed further adapting a protocol set for neurons [26]. Briefly, neurons plated on coverslips as previously described and were incubated with 100 μM MCB in recording buffer until a steady state was achieved. Images were acquired using a custom-made widefield imaging system built on an IX71 Olympus microscope equipped with a 20× water objective. A Xenon arc lamp with a monochromator was used for excitation set at 380 nm and collecting emitted light through a dichroic mirror 79001-ET Fura2 (Chroma) and a band-pass emission filter at 525/36 nm, using a Zyla CMOS camera (Andor). For image analysis, ROIs were then chosen and average MCB intensity for each neuron was calculated as an average of last three frames of the plateau and plotted in a scatter plot.

### Immunocytochemistry and confocal imaging

Neurons were plated on 96-well plates as previously described and grown until DIV12-14. Cells were then washed in PBS and fixed using 4% paraformaldehyde (P6148, Sigma) for 30 min at room temperature and then washed again. Blocking was performed by incubation with 3% Bovine Serum Albumin (A2153, Sigma) in PBS and staining using a primary antibody against an extracellular epitope of Grin2b (AGC-003, Alomone labs) and a secondary anti-rabbit Alexa488 (Thermo Fisher Scientific). Nuclei were stained with Hoechst 33342 and images were then collected by exciting Alexa488 at 488 nm and Hoechst at 405 nm, using a 60× objective and a Zeiss 880 confocal microscope.

### mRNA extraction and qPCR

mRNA was extracted from brain tissue using ReliaPrep™ RNA Cell Miniprep (Z6010, Promega); mRNA concentration was measured and 500 ng mRNA was used to obtain cDNA by means of the GoScript™ Reverse Transcription Kit (A5000, Promega), following manufacturer instructions.

The obtained cDNA was subjected to qPCR using SYBR^®^ Green JumpStart Taq ReadyMix (S4438, Sigma) and CFX96 Real-Time System (Biorad). Different pairs of primers where then used to quantify the mRNA expression levels of genes of interest:

Cyclophilin A F 5′-CCCACCGTGTTCTTCGACA-3′

Cyclophilin A R 5′-CCAGTGCTCAGAGCTCGAAA-3′

GRIK2 F5′-TGTGGAATCTGGCCCTATGG-3′

GRIK2 R5′-TGAACTGTGTGAAGGACCGA-3′

Grin2b F 5′-CGCCCAGATCCTCGATTTCA-3′

Grin2b R5′-CTGGAAGAACATGGAGGACTCA-3′

MCU F5′-GTCAGTTCACACTCAAGCCTAT-3′

MCU R 5′-TTGAAGCAGCAACGCGAACA-3′

MCURI F 5′-CTTCTGGGAGCAGGAAACTCTA-3′

MCURI R 5′-TGAGTAGCAAACCCATTGTC-3′

MICU1 F5′-GCTCCATAACGCCCAATGAG-3′

MICU1 R5′-GAAGGAGATGAGCCCACACT-3′

### Lipid extraction and lipidomics analysis

Lipids were extracted from brains (dissections performed on newborn pups, *n* = 3 per genotype) using Folch extraction (chloroform:methanol = 2:1) and were analyzed, as previously described [27]. In this configuration, measurements of GBA1 substrate are not able to differentiate between the combined glycosylceramides, and therefore include both glucosylceramides and galactosylceramides.

### Statistical analysis

Image quantification was performed using ImageJ and data were analyzed using GraphPad Prism. When the number of data points (usually corresponding to cell number) was >150 (*n* = 3 independent experiments), distribution analysis was performed (frequency distribution or cumulative frequency distribution), while when the number of data points was lower mean ± SD or mean ± SEM were used, representing single data points on graphs to properly account for data variability. Tests of normality were performed (Shapiro–Wilk test) to identify normal or non-normal populations. When normal, Student’s test or Anova tests (Bonferroni post-test) were used as needed, otherwise the nonparametric Kruskal–Wallis test (Dunns post-test) was used. The chosen tests are clearly indicated in the figure legends; *p < 0.05, **p < 0.01, and ***p < 0.001.

## Results

### *Gba1*^+/−^ and *gba1*^*−/−*^ neurons show delayed calcium deregulation in response to 10 µM glutamate concentrations

**Fig. 1:**
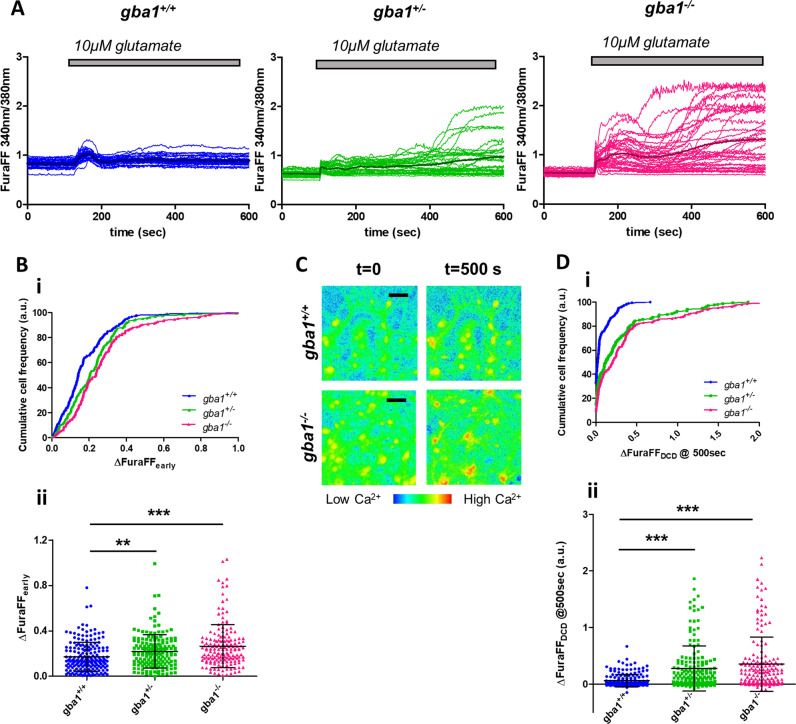
*Gba1*^+/−^ and *gba1*^*−/−*^ neurons show delayed calcium deregulation in response to low glutamate concentrations. **a** Neuronal cytosolic calcium concentration was measured by fluorescence imaging after labeling neurons with the low-affinity calcium sensor, FuraFF. Changes in [Ca^2+^]_c_ following exposure of neurons to 10 μM glutamate are plotted as a function of time for the different genotypes *gba1*^*+/+*^, *gba1*^*+/−*^, and *gba1*^*−/−*^, as indicated. The traces reveal a significant difference between the responses of each genotype upon 10 μM glutamate stimulation both in term of the immediate response, which was followed by delayed calcium deregulation (DCD) in a large proportion of *gba1*^*−/−*^ and a smaller but significant number of *gba1*^*+/−*^ cells (*n* = 3 independent experiments, *N* = 40–60 cells per genotype per experiment). **b** (i) The graphs show the cumulative frequency distribution of the peak values of the early response (at about 100 s) to 10 μM glutamate for the different genotypes and (ii) scatter plots of the peak values and mean ± SD. The data show a significant increase in [Ca^2+^]_c_ in the early response to glutamate in *gba1*^*+/−*^ or *gba1*^*−/−*^ compared with *gba1*^*+/+*^ (Kruskal–Wallis test, Dunns post-test, **p < 0.01 and ***p < 0.001, respectively). **c** FuraFF ratiometric images for *gba1*^*+/+*^ and *gba1*^*−/−*^ neurons (the image excited at 340 nm divided by that excited at 380 nm), shown at the start of the experiment (*t* = 0 s) and at 400 s after exposure to glutamate, showing that [Ca^2+^]_c_ had fully recovered in the control neurons, while the sustained very high [Ca^2+^]_c_ levels in the *gba1*^*−/−*^ neurons reflect deregulation of [Ca^2+^]_c_ homeostasis and DCD (scale bar = 25 µ). **d** (i) Cumulative frequency distribution of the peak values of DCD (400 s after stimulation) in response to 10 μM glutamate for the different genotypes and (ii) relative scatter plots of the peak values and mean ± SD. These data show the increased percentage of neurons showing DCD (ΔFuraFF > 0.1) in *gba1*^*+/−*^ or *gba1*^*−/−*^ cells compared with *gba1*^*+/+*^ (Kruskal–Wallis test, Dunns post-test, ***p < 0.001)

Mixed cultures of neurons and astrocytes were loaded with the low-affinity [Ca^2+^]_c_ indicator FuraFF-AM and images were acquired over time. Exposure to 10 μM glutamate (Fig. 1a) caused the expected rise in [Ca^2+^]_c_, but responses clearly differed between *gba1*^*+/+*^, *gba1*^*+/−*^, and *gba1*^*−/−*^ neurons. Both the early peak response, (ΔfuraFF_early_) and responses at later time points differed significantly between genotypes (Fig. 1bii and dii). Cumulative frequency distributions revealed a significant increase in the amplitude of the early glutamate response in both *gba1*^*−/−*^ and *gba1*^*+/−*^ neurons, compared with controls (Fig. 1bi, *n* = 3 mice per genotype, *N* = 30–60 cells per genotype per experiment). In the continued presence of glutamate, the initial transient response was followed in the majority of *gba1*^*−/−*^ cells by a delayed increase in [Ca^2+^]_c_, referred to as DCD, a characteristic response to toxic glutamate concentrations (100 μM or higher) in control cells [28, 29].

At 10 μM glutamate, DCD was seen in very few control cells, but was a feature of the majority of *gba1*^*−/−*^ cells, while *gba1*^*+/−*^ cells showed an intermediary response (Fig. 1a–c). The cumulative frequency distribution of the delayed response (ΔfuraFF_DCD_—Fig. 1di) and the scatter plot of the [Ca^2+^]_c_ response at 400 s after glutamate exposure (Fig. 1dii) indicated the percentage of neurons showing DCD (defined as ΔfuraFF > 0.1 ratio unit). In *gba1*^*+/−*^ and in *gba1*^*−/−*^ cultures, about 53 and 60% of the neurons showed DCD, respectively, while this was seen in only 20% of neurons in *gba1*^*+/+*^ cultures (Fig. 1d, *n* = 3 mice per genotype, *N* = 40–60 cells per genotype per experiment).

Neuronal responses to 1 μM glutamate, at the lower limit of the physiological range [30], also showed differences between genotypes consistent with these data (Supplementary Fig. 1A). [Ca^2+^]_c_ remained elevated 400 s after 1 μM glutamate in 10–15% of *gba1*^*+/−*^ and *gba1*^*−/−*^ neurons but only in 1% of *gba1*^*+/+*^ neurons.

In order to determine whether responses to Ca^2+^ signals arising from different sources also showed deregulation, we explored responses to release ER Ca^2+^ by metabotropic glutamate receptors and to electrical pacing, promoting Ca^2+^ influx through voltage-gated Ca^2+^ channels.

Ionotropic receptors were inhibited using 10 μM D-AP5 and 20 μM CNQX and neurons challenged with 100 μM quisqualate, a group I metabotropic receptor agonist [31]. Only very small responses were detectable using the low-affinity FuraFF (Supplementary Fig. 2A). Experiments with the higher affinity indicator Fura-2, revealed a small but significant reduction in the [Ca^2+^]_c_ peak response to quisqualate in *gba1*^*−/−*^ neurons (Supplementary Fig. 2B-C). Responses to electrical pacing were undetectable using FuraFF, while measurements with Fura-2 revealed no significant differences in peak responses between genotypes (Supplementary Fig. 2D). Altogether, these data show that DCD is a response only to Ca^2+^ influx through ionotropic pathways, probably reflecting the much greater Ca^2+^ load that this represents.

### Increased sensitivity of *gba1*^+/−^ and *gba1*^*−/−*^ neurons to glutamate is not due to different expression of glutamate receptors

**Fig. 2:**
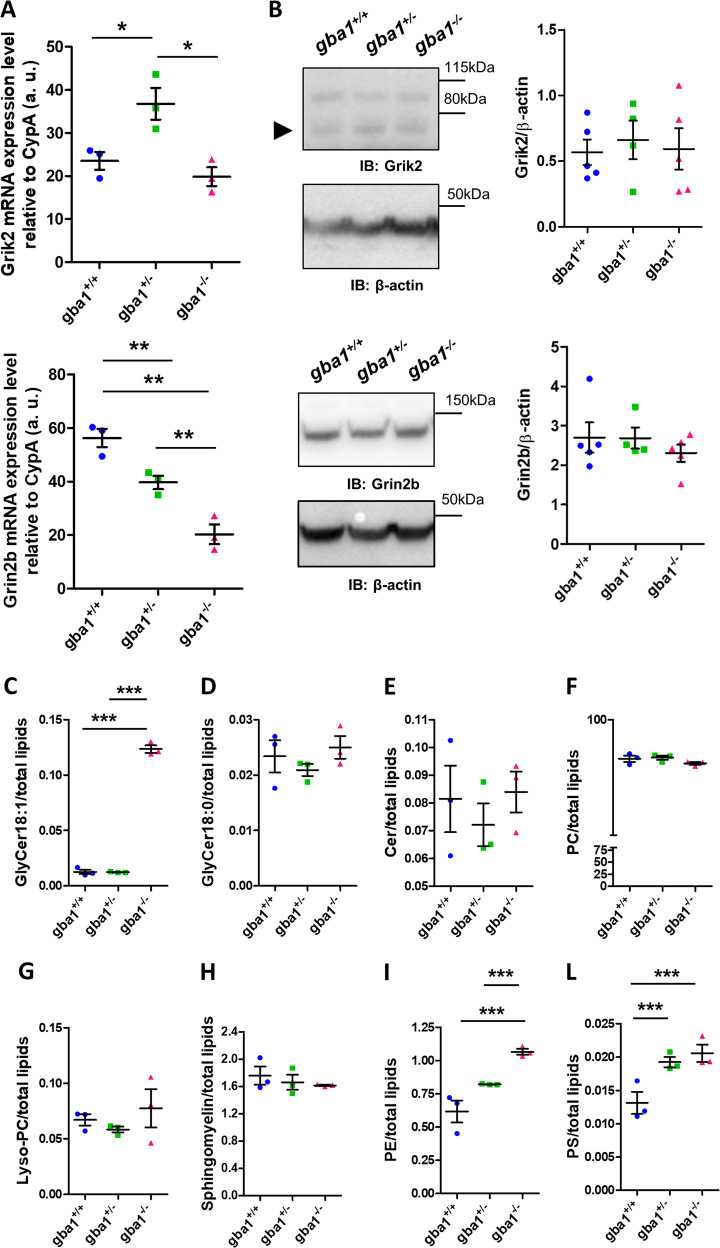
Levels of expression of glutamate receptors and massive accumulation of glycosylceramides are not responsible for increased sensitivity to glutamate in *gba1*^+/−^ and *gba1*^*−/−*^ neurons. **a** Expression of the glutamate receptors *Grik2* and *Grin2b* in *gba1*^*+/+*^, *gba1*^*+/−*^, and *gba1*^*−/−*^ mice brains was measured at the mRNA level by qPCR (shown as scatter plot, mean ± SEM, *n* = 3 brains per genotype). These data show that *Grik2* mRNA was slightly higher in *gba1*^*+/−*^ compared with the other genotypes, while *Grin2b* mRNA levels were decreased in *gba1*^*+/−*^ and *gba1*^*−/−*^ compared with *gba1*^*+/+*^, showing that the increased glutamate sensitivity is not attributable to increased glutamate receptor expression (One-way Anova, post-hoc Bonferroni, **p < 0.01). **b** Protein expression of the glutamate receptors *Grik2* and *Grin2b* in *gba1*^*+/+*^, *gba1*^*+/−*^, and *gba1*^*−/−*^ mice brains were measured by western blot (shown as scatter plot, mean ± SEM, *n* = 4–5 brains per genotype). These data show that the expression of these receptors was unchanged at the protein level (One-way Anova, post-hoc Bonferroni). **c**–**m** Analysis of lipidomics performed by mass spectrometry on lipids extracted from *gba1*^*+/+*^, *gba1*^*+/−*^, and *gba1*^*−/−*^ brains (*n* = 3 brains per genotype, data represented as scatter plot and mean ± SEM). Glycosylceramide (d18:1) levels were increased in *gba1*^*−/−*^ but not in *gba1*^*+/+*^ and *gba1*^*+/−*^ brains (One-way Anova, post-hoc Bonferroni, ***p < 0.001) (**c**), suggesting that the enzyme activity produced by a single copy of *GBA1* gene is sufficient to avoid substrate accumulation. Curiously, glycosylceramide (d18:0) levels were unchanged (**d**), as well as Cer, PC, lyso-PC, SM (**e**–**h**), while PE and PS are significantly changed in the disease models compared with wild-type brains (**i**–**l**)

The increased sensitivity to glutamate could reflect altered expression of ionotropic glutamate receptors. The ionotropic glutamate NMDA receptor subunit 2B (*Grin2b*) and the ionotropic kainate receptor subunit 2 (*Grik2*) are both lifespan modifier genes in GWAS studies of mouse strains treated with the GBA1 inhibitor, Conduritol B Epoxide (CBE) [32]. *Grin2b* mRNA expression is higher in mouse strains in which lifespan is shortened following CBE treatment, suggesting that expression of the glutamate receptor is increased as a secondary effect of GBA1 inhibition and sensitizes the cells. We therefore measured expression levels of mRNA for *Grin2b* and for *Grik2* in *gba1*^*+/+*^, *gba1*^*+/−*^, and *gba1*^*−/−*^ mouse brains by qPCR. mRNA levels of *Grik2* were slightly higher for *gba1*^*+/−*^ neurons compared with the other genotypes, while *Grin2b* mRNA levels showed a small but significant decrease in *gba1*^*+/−*^ and *gba1*^*−/−*^ compared with *gba1*^*+/+*^ (Fig. 2a). However, western blots of brain lysates to quantify Grik2 and Grin2b protein levels (Fig. 2b) (*n* = 4–5 per genotype) revealed no significant difference among genotypes. Surface analysis for Grin2b by immunofluorescence [33] and confocal imaging also failed to reveal any significant differences between them (Supplementary Fig. 3). Thus, the increased glutamate sensitivity and DCD is not a consequence of increased glutamate receptor expression.

As DCD is a predictor of neuronal cell death [34, 35], these data show that *gba1*^*−/−*^ and *gba1*^*+/−*^ neurons are more vulnerable to glutamate-induced [Ca^2+^]_c_ overload than the *gba1*^*+/+*^.

### Lipid homeostasis is differentially altered in the brain of *gba1*^*+/−*^ and *gba1*^*−/−*^ mice

The loss of GBA1 is expected to cause accumulation of its substrate glucosylceramide. Mass spectrometry analysis of lipids extracted from *gba1*^*+/+*^, *gba1*^*+/−*^, and *gba1*^*−/−*^ brains generates measurements of glycosylceramide, which corresponds to both glucosylceramide and galactosylceramide. The resulting data showed accumulation of glycosylceramides only in *gba1*^*−/−*^ but not in *gba1*^*+/−*^ brains, suggesting that one copy of the gene may generate sufficient enzyme to minimize substrate accumulation (Fig. 2b). However, it is important to emphasize that smaller increases in glucosylceramide levels in *gba1*^*+/−*^ brains may have been masked when measured together with galactosylceramide, because the latter are more abundant in subpopulations of cells in the brain. In fact, heterozygous GBA pure neurons may present glucosylceramide accumulation [36, 37]. Noteworthy, saturated glycosylceramides (d18:0) were not elevated even in *gba1*^*−/−*^ cells.

Interestingly, other secondary substrates may also be implicated. For example, glycosphingosine accumulation occurs in GD models and in patients with GD [38–40], and even if this does not seem to occur in *gba1*^*+/−*^ neurons [40], we cannot exclude a contribution from changes in overall lipid metabolism. Levels of ceramides, products of the GBA1 enzymatic activity, were not affected by the knockout, suggesting activation of compensatory pathways (Fig. 2e). Phosphatidylcholine (PC), lyso-PC, and sphingomyelin were also unaffected (Fig. 2f–h), while phosphatidylethanolamine (PE) and phosphatidylserine (PS), which are important for mitochondrial function and for regulation of autophagy [41–44], were both upregulated in *gba1*^*−/−*^ brains compared with *gba1*^*+/+*^ (Fig. 2i–l). PS was increased also in *gba1*^*+/−*^ brains, further suggesting that this pathway may contribute to the neuronal pathophysiology.

### Low glutamate concentrations cause loss of mitochondrial membrane potential in *gba1*^+/−^ and *gba1*^*−/−*^ neurons

The [Ca^2+^]_c_ increase in DCD associated with glutamate excitotoxicity is closely coupled to collapse of Δψm and attributed to impaired [Ca^2+^]_c_ homeostasis due to ATP depletion and the resultant failure of Ca^2+^ extrusion from the cytoplasm by Ca^2+^-H^+^ ATPases [29, 45], but this has not been demonstrated in a disease model.

**Fig. 3:**
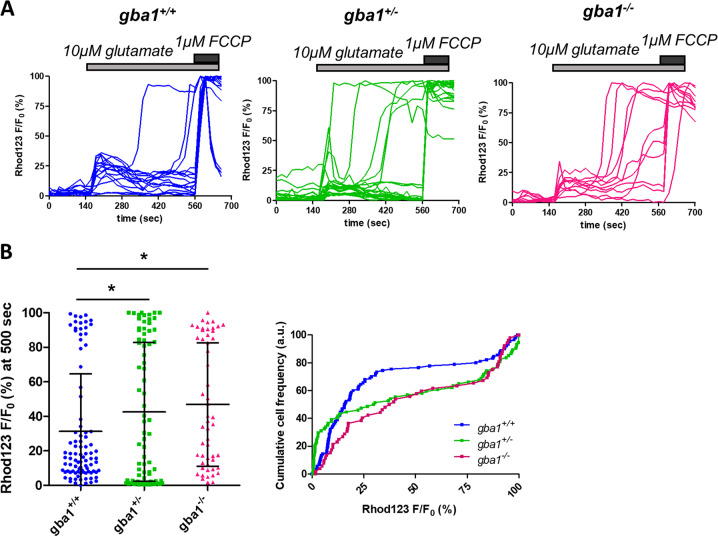
Low glutamate concentrations cause mitochondrial depolarization in *gba1*^+/−^ and *gba1*^*−/−*^ neurons. **a** The mitochondrial membrane potential was measured using rhodamine 123 (with the ‘dequench protocol’) in responses to exposure to 10 μM glutamate stimulation and plotted as a function of time for each genotype *gba1*^*+/+*^, *gba1*^*+/−*^, and *gba1*^*−/−*^. An increase in Rhod123 fluorescence intensity reports mitochondrial depolarization. The responses to 10 μM glutamate were significantly different between *gba1*^*−/−*^ and *gba1*^*+/+*^ or *gba1*^*+/−*^ genotypes, revealing a large depolarization in the *gba1*^*−/−*^ and in *gba*^*+/−*^ cells (*n* = 3 biological replicates, *N* = 15–25 cells per genotype per experiment). **b** Scatter plots of the mitochondrial depolarization at 360 s after 10 μM glutamate stimulation, expressed as Rhod123 F/F_0_ percentage comprised between the baseline (0%) and maximal fluorescence intensity value (100%) for the different genotypes. Average values are expressed as mean ± SD (left panel). Relative cumulative frequency distribution of the mitochondrial depolarization is also shown (right panel), showing a significant mitochondrial depolarization in response to 10 μM glutamate in *gba1*^*+/−*^ or *gba1*^*−/−*^ compared with *gba1*^*+/+*^ (Kruskal–Wallis test, Dunns post-test, *p < 0.05)

To further explore the relationship between mitochondrial (dys)function and DCD, we used Rhodamine123, in ‘dequench mode’ [46], to study time-dependent changes in Δψ_m_ following exposure to glutamate. After an initial transient depolarization, coincident with the initial [Ca^2+^]_c_ response to glutamate, Δψ_m_ recovered almost to the baseline in the majority of *gba1*^*+/+*^ neurons, while in *gba1*^*+/−*^ and *gba1*^*−/−*^ cells a large proportion of cells showed a delayed collapse of Δψ_m_ (Fig. 3a).

The secondary depolarization, quantified as the percentage change in the normalized Rhodamine123 signal at 400 s after glutamate stimulation (see methods) was significantly different between genotypes (Fig. 3b): only 23% of the *gba1*^*+/+*^ neurons presented at least 50% change of signal 400 s after 10 μM glutamate stimulation, while an equivalent depolarization was seen in 42% of the *gba1*^*+/−*^ and 43% of the *gba1*^*−/−*^ neurons, closely resembling the distribution of the DCD responses.

These measurements confirm the close coupling between collapse of Δψ_m_ and DCD that we have previously described in other models [29].

### Increased sensitivity to glutamate reflects impaired capacity to maintain ATP homeostasis in *gba1*^+/−^ and *gba1*^*−/−*^ neurons

The appearance of DCD in response to low glutamate concentrations strongly resembles the glutamate response of neurons following inhibition of oxidative phosphorylation by oligomycin [34, 45, 47] suggesting that glutamate-induced DCD reflects bioenergetic insufficiency in *gba1*^*+/−*^ and *gba1*^*−/−*^ neurons. We therefore measured dynamic changes in neuronal ATP:ADP ratios in response to glutamate.

**Fig. 4:**
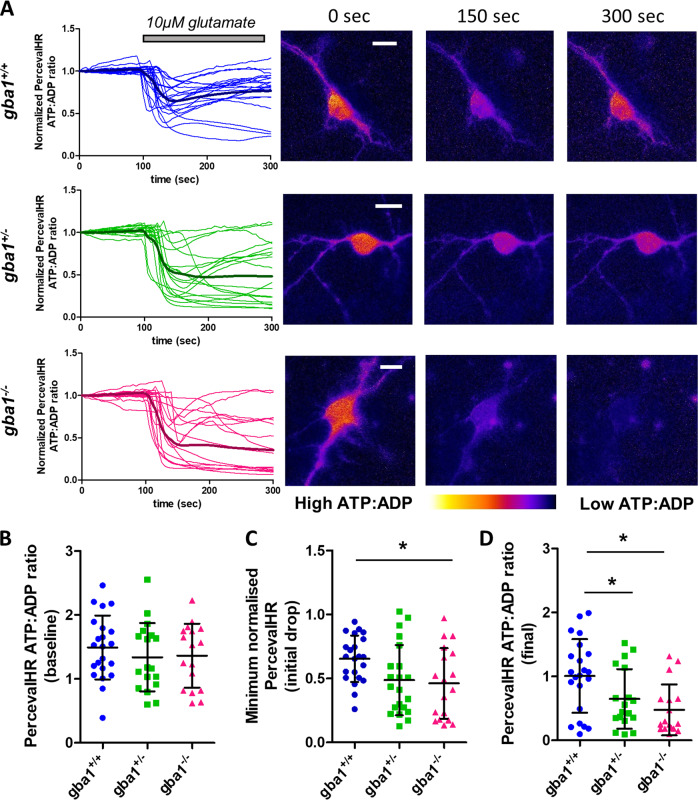
Low glutamate concentrations cause an irreversible fall of ATP:ADP ratio in *gba1*^+/−^ and *gba1*^*−/−*^ neurons. **a** The ATP:ADP ratio was measured at the level of single cells using the genetically encoded reporter, PercevalHR, and normalized to the baseline of each response. The PercevalHR ratio following exposure of cells to 10 μM glutamate is plotted as a function of time, for each genotype *gba1*^*+/+*^, *gba1*^*+/−*^, and *gba1*^*−/−*^. Corresponding ratiometric images for three different time points (0, 150, and 300 s) are also shown (scale bar = 10μm). 10 μM glutamate caused a rapid reduction in ATP:ADP, which largely recovered in the majority of *gba1*^*+/+*^ cells but failed to recover in *gba1*^*+/−*^ and *gba1*^*−/−*^ cells. **b** Scatter plot and mean ± SD of ATP:ADP ratio in basal conditions (calculated from the raw, non-normalized signals) for the different genotypes *gba1*^*+/+*^, *gba1*^*+/−*^, and *gba1*^*−/−*^ show no difference at the basal level (Kruskal–Wallis test, Dunns post-test). **c** Scatter plot and mean ± SD of ATP:ADP ratio immediately after exposure of cells of each genotype to 10 μM glutamate, expressed as the minimum normalized ATP:ADP ratio after stimulation, show that the initial drop in ATP:ADP ratio in *gba1*^*−/−*^ cells was significantly greater than the drop in *gba1*^*+/+*^ neurons (Kruskal–Wallis test, Dunns post-test, *p < 0.05). **d** Scatter plot and mean ± SD of ATP:ADP ratio 200 s after exposure of cells of each genotype to 10 μM glutamate (as calculated starting from nonnormalized signals) show that ATP:ADP ratio largely recovered in *gba1*^*+/+*^ cells but failed to recover in both *gba1*^*+/−*^ and *gba1*^*−/−*^ neurons (Kruskal–Wallis test, Dunns post-test, *p < 0.05)

Neurons were transfected with the ratiometric fluorescent probe PercevalHR, which reports changes in cytosolic ATP:ADP [24]. Most neurons responded to glutamate with a rapid decrease in the ATP:ADP ratio (Fig. 4a), reported by the probe as a decrease in the ATP sensitive signal and an increase in the ADP sensitive signal (Supplementary Fig. 4). The ATP:ADP ratio recovered over a few minutes in most control cells, but recovery was much slower or absent in the *gba1*^*−/−*^ and to a lesser extent in the *gba1*^*+/−*^ neurons.

Quantifying the ATP:ADP ratios before glutamate exposure, immediately after and 200 s after 10 µM glutamate stimulation (Fig. 4b–d), showed that there were no significant differences between basal ATP:ADP ratios (before normalization) among the different genotypes (Fig. 4b), and the initial drop in ATP:ADP ratio was significantly higher in *gba1*^*−/−*^ neurons compared with wild-type (Fig. 4c). Moreover, recovery to the baseline was markedly impaired in both *gba1*^*+/−*^ and *gba1*^*−/−*^ cells (Fig. 4d).

Interestingly, the same experiments in control neurons in response to toxic concentrations of glutamate (100 μM) (Supplementary Fig. 5), showed an initial decrease in the ATP:ADP ratio, followed by a partial recovery before undergoing a secondary decrease. This behavior was quite distinct from the responses seen in *gba1*^*+/−*^ and *gba1*^*−/−*^ neurons upon 10 μM glutamate stimulation. However, when control neurons were treated first with 1 μM oligomycin, to inhibit oxidative phosphorylation, their responses to 10 μM glutamate resembled the responses of the *gba1*^*−/−*^ neurons, showing a decrease that failed to recover. These data further suggest that ATP depletion in *gba1*^*+/−*^ and *gba1*^*−/−*^ neurons in response to nontoxic glutamate concentrations is a consequence of impaired mitochondrial function.

These data suggest that even though energy homeostatic mechanisms maintain a normal ATP:ADP ratio at rest, the underlying loss of mitochondrial bioenergetic capacity undermines the possibility to match ATP production to meet the increased energy demand following glutamate stimulation.

### Mitochondrial calcium uptake is reduced in *gba1*^*−/−*^ and in *gba1*^*+/−*^ neurons

**Fig. 5:**
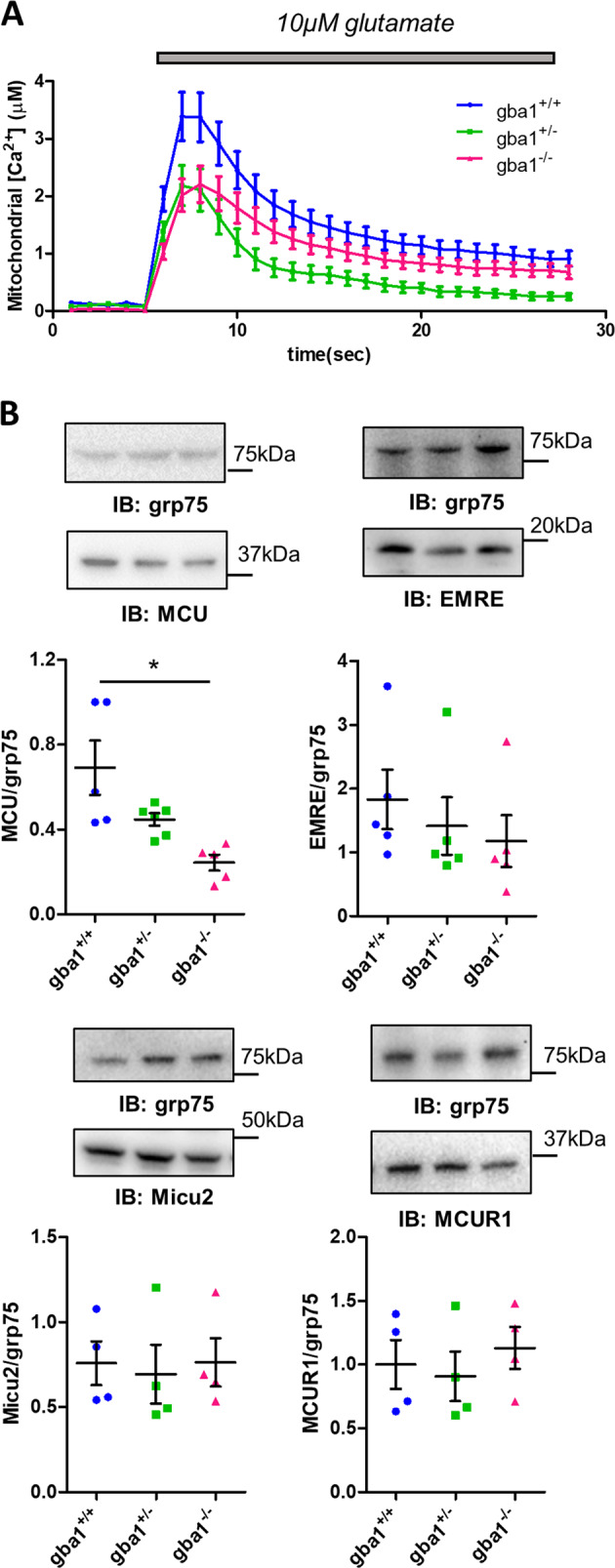
Mitochondrial calcium buffering capacity is reduced and MCU protein level is decreased in *gba1*^*−/−*^ and *gba1*^*+/−*^ neurons. **a** Mitochondria-target aequorin plate reader assay was used to measure mitochondrial Ca^2+^ uptake in mixed neuronal and astrocytic cultures from *gba1*^*+/+*^, *gba1*^*+/−*^, and *gba1*^*−/−*^ in response to 10 μM glutamate. The data show that mitochondrial Ca^2+^ uptake was significantly reduced in both *gba1*^*+/−*^ and *gba1*^*−/−*^ cells compared with *gba1*^*+/+*^ (*n* = 3–5 cultures per genotype, one-way Anova, post-hoc Bonferroni). **b** Protein expression levels of MCU complex components and MCU regulatory proteins evaluated by western blot in *gba1*^*+/+*^, *gba1*^*+/−*^, and *gba1*^*−/−*^ brains (data shown as scatter plots and mean ± SEM, *n* = 3–5 brains per genotype). MCU expression was significantly downregulated in *gba1*^*+/−*^ and *gba1*^*−/−*^ tissue (One-way Anova, post-hoc Bonferroni, *p < 0.05), while EMRE, MICU2, and MCUR1 expression levels were unchanged

The increased demand imposed on the cell by a [Ca^2+^]_c_ signal may be matched by an increased energy supply driven by the upregulation of the mitochondrial citric acid cycle in response to a rise in intramitochondrial Ca^2+^ concentration ([Ca^2+^]_m_) [48–51]. We therefore measured changes in [Ca^2+^]_m_ in response to 10 μM glutamate directly using mitochondria-targeted aequorin (Fig. 5a). Surprisingly, mitochondrial Ca^2+^ uptake was significantly reduced in both *gba1*^*−/−*^ and *gba1*^*+/−*^ neurons compared with *gba1*^*+/+*^.

This is especially significant as the initial transient increase in cytosolic [Ca^2+^] in response to glutamate was increased in the *gba1*^*+/−*^ and *gba1*^*−/−*^ cells (see above, Fig. 1b), consistent with impaired mitochondrial Ca^2+^ buffering. Resting cytosolic Ca^2+^ levels were not significantly different between populations, suggesting that the bioenergetic defect was not severe enough to impair resting Ca^2+^ homeostasis (Supplementary Fig. 6).

These findings may be attributable to the reduction in Δψ_m_ seen in *gba1*^*−/−*^ and *gba1*^*+/−*^ neurons [11]. However, we also explored the expression levels of the components of the mitochondrial Ca^2+^ uniporter complex, which may also contribute to altered mitochondrial Ca^2+^ uptake [17, 19, 52–54]. MCU, EMRE, MICU2, and MCUR1 protein levels were measured by western blot in brains from *gba1*^*+/+*^, *gba1*^*+/−*^, and *gba1*^*−/−*^ mice (Fig. 5b). Quantification showed that MCU expression was significantly reduced in the *gba1*^*+/−*^ and *gba1*^*−/−*^ cells, while expression levels of the associated regulatory proteins, EMRE, MICU2, and MCUR1 were not altered (*n* = 3–5 per genotype).

Quantification of mRNA for MCU, EMRE, MICU1, and MCUR1 by qPCR (Supplementary Fig. 7) did not reveal any significant differences (*n* = 3 per genotype), suggesting that changes in MCU expression must be post transcriptionally regulated.

### Rates of free radical production are increased in *gba1*^*−/−*^ neurons

**Fig. 6:**
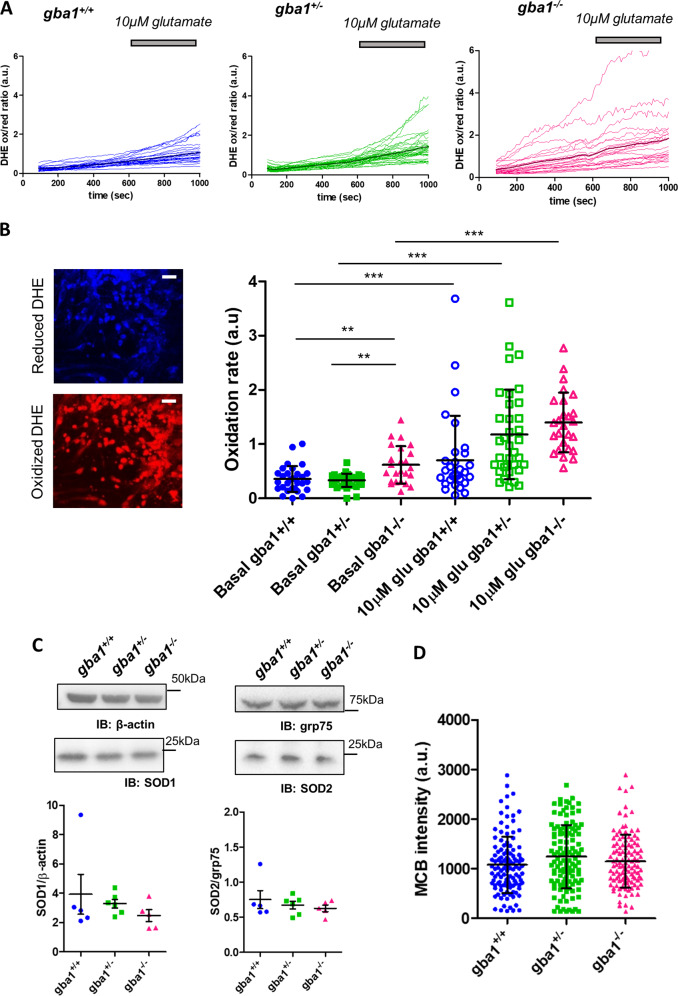
Rates of free radical production are increased in *gba1*^*−/−*^ neurons. **a** The rate of oxidation of Dihydroethidium (DHE) to a red fluorescent product was used to measure the rate of free radical generation with time in live cells. This was calculated as the intensity ratio between the oxidized (red) and the reduced (blue) forms DHE (shown as red and blue in the fluorescence images, respectively), under basal conditions and after 10 μM glutamate stimulation. **b** Representative confocal images of oxidized and reduced DHE in cells (scale bar = 25 μm). Representative DHE oxidation rates quantification as measured from the slopes of the curves showing DHE oxidation over time, before and after 10 μM glutamate stimulation for *gba1*^*+/+*^, *gba1*^*+/−*^, and *gba1*^*−/−*^ cells (data shown as scatter plots and mean ± SD, *n* = 3 independent experiments, *N* = 15–30 cells per genotype per experiment, Kruskal–Wallis test, Dunns post-test, **p < 0.01 and ***p < 0.001). **c** Western blot analysis of the expression levels of antioxidant proteins SOD1 and SOD2 (data shown as scatter plots and mean ± SEM, *n* = 5–6 brains per genotype) normalized to beta-actin and grp75, respectively. The scatter plot showing the result for protein expression suggests that there was no significant difference among the different genotypes (One-way Anova, post-hoc Bonferroni). **d** Glutathione levels were measured by imaging MCB intensity for neurons from each of the three genotypes (data shown as scatter plots and mean ± SD, *n* = 3 independent experiments, *N* = 35–40 per genotype per experiment). No significant difference was observed (Kruskal–Wallis test, Dunns post-test)

Ca^2+^-dependent neuronal injury may be exacerbated by the conjunction of raised [Ca^2+^]_m_ with oxidative stress [23]. We therefore used dihydroethidium (DHE), a ratiometric fluorescent reporter sensitive to reactive oxygen species (ROS) to determine whether basal rates of free radical production differ in *gba1*^*−/−*^ cells (Fig. 6a) [55]. These data showed an increased basal rate of free radical production in *gba1*^*−/−*^ cells compared with the other genotypes (Fig. 6b). Exposure of cells to 10 μM glutamate caused a significant increase in the rate of ROS generation in each genotype, but the relative change was not significantly different between *gba1*^*+/+*^, *gba1*^*+/−*^, and *gba1*^*−/−*^ cultures (Fig. 6b) (*n* = 3 independent experiments, *N* = 10–20 cells per genotype per experiment).

To address the possibility that the increased rate of resting free radical production in *gba1*^*−/−*^ was associated with impaired antioxidant defenses, we measured the expression of superoxide dismutases (SODs) and glutathione (GSH) levels. Western blots of cytosolic SOD1 and mitochondrial SOD2 showed no difference between genotypes (*n* = 5 per genotype) (Fig. 6c). GSH levels were measured using monochlorobimane (MCB) as previously described [26]. MCB reacts with GSH, generating a fluorescent adduct, so a measure of steady-state MCB intensity gives a measure of relative GSH content. MCB intensity in *gba1*^*+/+*^, *gba1*^*+/−*^, and *gba1*^*−/−*^ showed no significant difference between genotypes (Fig. 6d—*n* = 3 independent experiments, *N* = 35–40 cells per genotype per experiment).

## Discussion

The goal of this study was to evaluate the functional consequences of mitochondrial dysfunction on cell physiology in neurons from the transgenic *gba1*^*−/−*^ mouse, a model for severe neuropathic GD, and from the *gba1*^*+/−*^ mouse, which may illuminate mechanisms of neurodegeneration in GBA1-related PD. The functional impact of impaired mitochondrial function is most dramatically revealed in neurons by an impaired capacity to respond to dynamic changes in metabolic demand, such as the increased energy drain imposed by exposure to glutamate. Exposure of the cells to glutamate at concentrations that are innocuous for wild-type cells caused a profoundly dysregulated response in terms of [Ca^2+^]_c_ signaling and mitochondrial metabolism in the *gba1*^*−/−*^ and, to a lesser extent, in the *gba1*^*+/−*^ neurons.

The *gba1*^*−/−*^ mice suffer from an aggressive form of neurodegeneration, and die only 2 weeks after birth [12], while *gba1*^*+/−*^ mice do not show a disease-related phenotype. However, neurons from both *gba1*^*+/−*^ and *gba1*^*−/−*^ show a significant decrease in Δψ_m_ and *gba1*^*−/−*^ mixed cultures of neurons and astrocytes also show reduced basal respiratory activity and massively reduced maximal respiratory capacity [11]. We found that neurons from both *gba1*^*−/−*^ and *gba1*^*+/−*^ mice showed abnormal responses to 10 μM glutamate, characterized as DCD, which is normally associated with ‘excitotoxicity’ in response to much higher concentrations of glutamate. We attribute this vulnerability primarily to the decreased bioenergetic capacity, which is especially severe in the *gba1*^*−/−*^ neurons [11], and therefore to the failure to maintain ATP homeostasis in the face of increased energy demand imposed by glutamate.

The decreased mitochondrial Ca^2+^ uptake that we have shown in both *gba1*^*−/−*^ and *gba1*^*+/−*^ neurons, will likely contribute to this energetic failure, as it will limit the capacity of the mitochondria to increase ATP production in response to [Ca^2+^]_c_ signals. The observed reduction in mitochondrial Ca^2+^ uptake is attributable to the reduced Δψ_m_ but also to the reduced expression of the MCU protein, which seems to be associated with changes in protein turnover rather than to transcriptional repression.

The small reduction of *Grin2b* mRNA in both *gba1*^*+/−*^ and *gba1*^*−/−*^ brains may represent another compensatory mechanism, but was not reflected in changes in Grin2b protein expression, or in the localization of Grin2b at the plasma membrane. Thus, changes in Grin2b expression or localization are unlikely to be responsible for DCD. Furthermore, responses of *gba1*^*−/−*^ neurons to release of ER Ca^2+^ by metabotropic receptor activation showed a modest reduction, suggesting that the key Ca^2+^ source that triggers DCD is delivered through ionotropic glutamate receptors.

The response to the Ca^2+^ influx is likely compounded by an increased rate of ROS generation in *gba1*^*−/−*^ neurons under basal conditions, as glutamate toxicity is exacerbated by oxidative stress [56].

Interestingly, we have found a marked cellular phenotype in *gba1*^*+/−*^ neurons. We previously showed, that Δψ_m_ is reduced in *gba1*^*+/−*^ neurons [11], although defects in respiratory capacity were less severe than in homozygotes. These differences, together with the difference in oxidative stress, that was increased only in *gba1*^*−/−*^ neurons, may contribute to the difference of disease phenotype in the *gba1*^*−/−*^ and *gba1*^*+/−*^ mice. In agreement with our data, a mouse model carrying the heterozygous *GBA1* PD-associated mutation L444P was shown to have defective mitochondria, supporting a role of impaired bioenergetics in GBA1-associated PD [57]. Dopaminergic neurons at risk of neurodegeneration in PD are physiologically characterized by Ca^2+^-dependent pace-making activity, while intrinsic Ca^2+^ buffering capacity is reduced [22], imposing a major energy demand, which will be amplified by mechanisms that compromise bioenergetic reserve, putting these cells especially at risk [58]. Since we have shown that partial depletion of GBA1 in *gba1*^*+/−*^ neurons sensitizes neurons to Ca^2+^ influx and show DCD in response to physiological glutamate concentrations, we suggest that the compromised mitochondrial function in these cells may increase the risk of neurodegeneration in neurons that are already vulnerable because of their normal physiological activity.

It is notable that both *gba1*^*−/−*^ and *gba1*^*+/−*^ neurons showed similar responses to glutamate and impaired [Ca^2+^]_c_ handling, suggesting that it is unlikely that the metabolic and signaling defects are simply attributable to the massive accumulation of the GBA1 substrate glucosylceramide, observed in *gba1*^*−/−*^, which was evident despite the presence of galactosylceramides. However, subtle changes of glucosylceramide levels in *gba1*^*+/−*^ brains may have been masked by the higher levels of galactosylceramides in the mixed cells. Considering this and the deregulation observed in PE and PS levels in *gba1*^*+/−*^ and *gba1*^*−/−*^ brains, broader evaluation of lipid homeostasis may help in understanding the pathophysiological mechanisms that couple reduced GBA1 to mitochondrial dysfunction.

Overall, our findings suggest that *gba1*^*−/−*^ but also *gba1*^*+/−*^ neurons are sensitive to dynamic changes in energy demand caused by an imposed workload, while under basal conditions, both ATP and cytosolic Ca^2+^ levels in *gba1*^*−/−*^ and *gba1*^*+/−*^ neurons were not different from the control. This implies the activity of a vicious cycle in which every mechanism enlisted to compensate a homeostatic Ca^2+^ stress, an increased energy demand and oxidative stress cause further deterioration of cell bioenergetic capacity, triggering a pathological cascade.

Our data highlight a general principle—that in any disease (from age-related neurodegenerative diseases to lysosomal storage disorders) in which mitochondrial bioenergetic capacity is impaired, neurons will become more vulnerable to increased energy demand, which may be sufficient to initiate dysregulated calcium signaling and cell death.

## Supplementary information

Author contribution to the article
