# Correction: Impaired cellular bioenergetics caused by GBA1 depletion sensitizes neurons to calcium overload

**DOI:** 10.1038/s41418-020-0525-0

**Published:** 2020-03-09

**Authors:** Nicoletta Plotegher, Dany Perocheau, Ruggero Ferrazza, Giulia Massaro, Gauri Bhosale, Federico Zambon, Ahad A. Rahim, Graziano Guella, Simon N. Waddington, Gyorgy Szabadkai, Michael R. Duchen

**Affiliations:** 1 Cell and Developmental Biology Department, University College London, London, WC1E6XA UK; 2 Institute for Women’s Health, University College London, London, WC1E6HU UK; 3 Department of Physics, University of Trento, 38123 Povo, TN Italy; 4 School of Pharmacy, University College London, London, WC1N1AX UK; 5 MRC Antiviral Gene Therapy Research Unit, Faculty of Health Sciences, University of the Witwatersrand, Johannesburg, South Africa; 6 Department of Biomedical Sciences, University of Padua, 35131 Padua, Italy; 7 The Francis Crick Institute, London, NW1 1AT UK; 8 Present Address: Department of Biology, University of Padua, 35131 Padua, Italy

**Keywords:** Neuroscience, Physiology, Neurological disorders

**Correction to: Cell Death & Differentiation**

10.1038/s41418-019-0442-2

This article was originally published under a non-CC-BY licence, but has now been made available under a CC-BY 4.0 license.

The PDF and HTML versions of the paper have been modified accordingly.

