# Supplementary material for: Impaired cellular bioenergetics caused by GBA1 depletion sensitizes neurons to calcium overload
Source: Cell Death Differ. 2019 Nov 4;27(5):1588–603. doi: 10.1038/s41418-019-0442-2 (PMC7206133; doi:10.1038/s41418-019-0442-2)
Supplement: Supplementary file 1 — Author contribution to the article [file 41418_2019_442_MOESM1_ESM.pdf]

DECLARATION OF CONTRIBUTIONS TO ARTICLE

ADMC

Manuscript Number: CDD-19-0176RR

Journal Name: *Cell Death & Differentiation* (the 'Journal')

Proposed Title of the Contribution:

Impaired cellular bioenergetics caused by GBA1 depletion sensitises neurons to calcium overload (the 'Contribution)

Author(s):

N. Plotegher, D. Perocheau, R. Ferrazza, G. Massaro, G. Bhosale, F. Zambon, A. A. Rahim, G. Guella, S. N. Waddington, G. Szabadkai, M. R. Duchen (the 'Authors')

For all *CDD* articles, each person named as an author in the published version must be able to show he or she has contributed substantially to the article.

Authorship credit should be based on 1) substantial contributions to conception and design, acquisition of data, or analysis and interpretation of data; 2) drafting the article or revising it critically for important intellectual content; and 3) final approval of the version to be published. Authors should meet conditions 1, 2 and 3.

Any person who cannot be shown to have made a substantial contribution to the article cannot be listed as an author in the final version. The name of any person who is deemed to have made a minor contribution can, however, appear in the Acknowledgments section of the article.

Please complete the table below to indicate the contributions of all named authors to the manuscript.

Author Full Name:	Specification of Contribution to the Manuscript:
Nicoletta Plotegher	Designed and conducted the experiments and wrote the paper. She approved the final version.
Dany Perocheu	Mouse colony work and genotyping. He critically revised the paper and approved the final version.
Ruggero Ferrazza	He performed all the mass spectrometry experiments and data analysis. He critically revised the paper and approved the final version.
Giulia Massaro	Mice colony work and genotyping. She critically revised the paper and approved the final version.
Gauri Bhosale	She performed immunofluorescence and imaging experiments. She critically revised the paper and approved the final version.
Federico Zambon	Performed calcium imaging experiments and western blot. He critically revised the paper and approved the final version.
Ahad A. Rahim	He established the mouse colony and contributed to the conception of the experimental design. He critically revised the paper and approved the final version.
Graziano Guella	He concieved the mass spectrometry analysis experiments, and he critically revised the paper and approved the final version.
Simon N. Waddington	He established the mouse colony and ensured availability of animals for us to us, and he contributed to the conception of the experimental design. He critically revised the paper and approved the final version.
Gyorgy Szabadkai	He contributed to the design and interpretation of the calcium experiments. He critically revised the paper and approved the final version.
Michael R. Duchen	He conceived the project, wrote the grant, designed the experiments and wrote the paper. He approved the final version.

Please complete the table below to indicate the contributions of all named authors to the figures.

Figure 1:

Nicoletta Plotegher, Federico Zambon, Dany Perocheu, Giulia Massaro, Ahad A. Rahim, Simon N. Waddington, Gyorgy Szabadkai, Michael Duchen

Figure 2:

Nicoletta Plotegher, Dany Perocheu, Giulia Massaro, Federico Zambon, Graziano Guella, Ruggero Ferrazza, Michael Duchen

Figure 3:

Nicoletta Plotegher, Dany Perocheu, Giulia Massaro, Michael Duchen

Figure 4:

Nicoletta Plotegher, Gauri Bhosale, Dany Perocheu, Giulia Massaro, Gyorgy Szabadkai, Michael Duchen

Figure 5:

Nicoletta Plotegher, Dany Perocheu, Giulia Massaro, Gyorgy Szabadkai, Michael Duchen

Figure 6:

Nicoletta Plotegher, Dany Perocheu, Giulia Massaro, Ahad A. Rahim, Simon N. Waddington, Gyorgy Szabadkai, Michael Duchen

Signed for and on behalf of the Author(s):

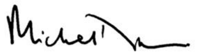

Print Name: Michael R. Duchen

Date: 12/09/2019
